# Lenalidomide and Low Dose Dexamethasone Plus Elotuzumab or Carfilzomib for Relapsed or Refractory Multiple Myeloma: A Comparison of Progression-Free Survival with Reconstructed Individual Participant Data

**DOI:** 10.1155/2018/9057823

**Published:** 2018-12-16

**Authors:** Shuo Li, Xiang-Yu Meng, Souraka Tapara Dramani Maman, Yong-Nong Xiao, Sheng Li

**Affiliations:** ^1^Department of Laboratory Medicine, Clinical Laboratory Medicine and Center for Gene Diagnosis, Zhongnan Hospital of Wuhan University, Wuhan 430071, China; ^2^Department of Urology, Zhongnan Hospital of Wuhan University, Wuhan 430071, China; ^3^Department of Hematology, Zhongnan Hospital of Wuhan University, Wuhan 430071, China; ^4^Department of Biological Repositories, Zhongnan Hospital of Wuhan University, Wuhan 430071, China

## Abstract

**Background:**

Refractory and relapsed multiple myeloma (RRMM) remains a clinical challenge. We compared the progression-free survival (PFS) of RRMM patients treated with lenalidomide and low dose dexamethasone plus elotuzumab or carfilzomib (ELD vs. CLD), using reconstructed individual patient data (IPD) based on two published trials reports.

**Methods:**

We extracted data of study-level characteristics from original trial reports. We evaluated the comparability between the two treatment groups in terms of baseline status. Digitization of PFS Kaplan-Meier curves, reconstruction of IPD data, and subsequent survival analysis were performed. Distribution of progression and death events over time was visualized as histograms and corresponding kernel density lines, and Kaplan-Meier survival curves were plotted. Hazard ratio (HR) and corresponding 95% confidence interval (95% CI) were calculated.

**Results:**

Significant difference in race and disease stage distribution was found (P < 0.0001). Higher proportion of white patients and patients with advanced disease in the carfilzomib group was identified. Survival analysis revealed better PFS in the carfilzomib group (elotuzumab group vs. carfilzomib group: HR = 1.36, 95% CI = [1.11-1.67]).

**Conclusion:**

The CLD regimen may result in better PFS as compared with the ELD regimen in RRMM patients.

## 1. Introduction

Since the introduction of novel drugs such as proteasome inhibitors (PIs) and immunomodulatory drugs (IMiDs), evident progress has been achieved in the management of multiple myeloma (MM) [1, 2]. However, it is still an incurable malignancy with increasing burden of disease [3]. Refractory and relapsed MM (RRMM) resistant to prior treatments is an important clinical challenge [4, 5]. RRMM patients form a heterogeneous group with diverse response to certain treatment regimen and poor prognosis [4]. Therefore, this subgroup is always the initial recipient of newly developed treatment regimens tested in clinical trials.

However, participating in a randomized clinical trial (RCT) does not promise a better outcome, and each participant is equally subjected to the risk of being assigned to a possibly ineffective intervention. This practical and ethical dilemma has haunted over trial participants ever since the first clinical trial was performed [6]. On the other hand, although the RCT is considered as the best approach to generating high quality evidence, its application is limited by its considerable financial and ethical cost.

Secondary analysis based on RCTs can provide useful information for healthcare professionals [7]. Recently, a method for reconstructing individual participant data (IPD) using digitized Kaplan-Meir curve data has been reported [8]. As a result, a comparison on survival outcomes of arms from different trials is made possible, under the condition that the baseline characteristics of patients are generally comparable among the trials. Findings revealed by this method may provide valuable information and save the cost of a direct comparison in additional trials.

Recently published RCTs of relapsed myeloma or RRMM mainly focused on new regimens containing latest drugs for patients with relapse or drug resistance after prior lines of treatment [9–20]. After a systematic literature review of these RCTs, we found that the treatment efficacy of two novel regimens, i.e., elotuzumab or carfilzomib plus lenalidomide and low dose dexamethasone (ELD and CLD regimen), has not yet been directly compared in clinical trials. Two previous Phase 3 RCTs have, respectively, compared the treatment effects of ELD or CLD versus the backbone lenalidomide and low dose dexamethasone and provided in the trial reports high quality survival curves of progression-free survival (PFS) [19, 20], based on which individual participant data (IPD) can be inferred using Guyot et al.'s method and used for subsequent survival analysis [8]. Therefore, we accordingly performed this cross-trial secondary analysis comparing the PFS of RRMM patients treated with ELD and CLD, as a comparison of treatment efficacy for the two regimens, in order to provide additional information for clinical practice and future study design.

## 2. Materials and Methods

Data on basic information of the original two RCTs and baseline characteristics of these two patient groups were extracted from original reports and carefully evaluated [19, 20]. For categorical data, the chi-square test was used to detect significant difference between these two groups. For continuous variables including age and time since diagnosis and number of prior treatments and since neither the mean and standard deviation nor patient-level data were provided in the original reports, we could not perform any statistical test to detect significant difference. A *P* < 0.05 indicated statistical significance.

To reconstruct PFS data of individual patients, methods developed by Guyot et al. were used [8]. Digitization of Kaplan-Meier curves was performed using plot digitizer 2.6.6 for windows. Reconstruction of survival data and subsequent survival analysis and visualization were performed using R 3.2.1 for windows. Distribution of progression and death events over time was visualized as histograms and corresponding kernel density lines, and Kaplan-Meier (KM) survival curves were plotted, with median PFS calculated for each group [21]. Median PFS obtained with reconstructed IPD was compared with median PFS provided in the original studies to examine the accuracy of the algorithm and reliability of reconstructed IPD. Hazard ratio (HR) and corresponding 95% confidence interval (CI) were calculated using the Cox proportional hazard model [21]. A 95% CI not covering one indicated statistically significant difference. Possible influence of any baseline difference on the interpretation of PFS results was further discussed.

## 3. Results

Regimens and baseline characteristics of included treatment arms are shown in Tables 1 and 2, respectively. According to results of chi-square test, the proportion of white patients was significantly higher in the carfilzomib group than the elotuzumab group (*P* < 0.0001). Distribution of disease stage was significantly different between these two groups (stage I disease: 44% vs. 16%, 32% vs. 25%, 21% vs. 47%, respectively, in the elotuzumab group and carfilzomib group for stage I, II, and III, *P* < 0.0001), indicating more advanced cases in the carfilzomib group. No significant difference was found regarding gender, Eastern Cooperative Oncology Group (ECOG) performance, and number of previous therapy received between these two groups (*P* = 0.16, 0.08 and 0.96, respectively). Another obvious difference is that the elotuzumab trial included both refractory and relapsed patients (the exact proportions unknown), and the carfilzomib trial included only relapsed patients.

**Figure 1:**
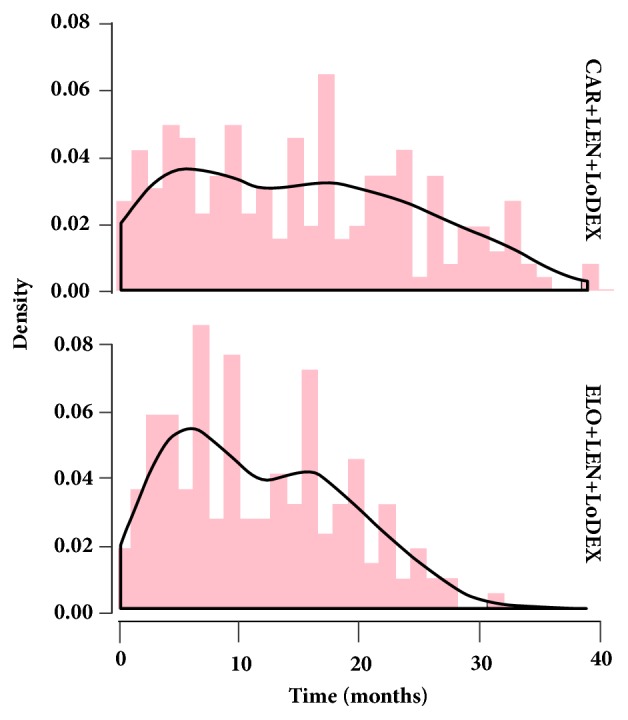
Histogram and kernel density of event distribution over time based on reconstructed individual-patient data for the carfilzomib and elotuzumab group.

**Figure 2:**
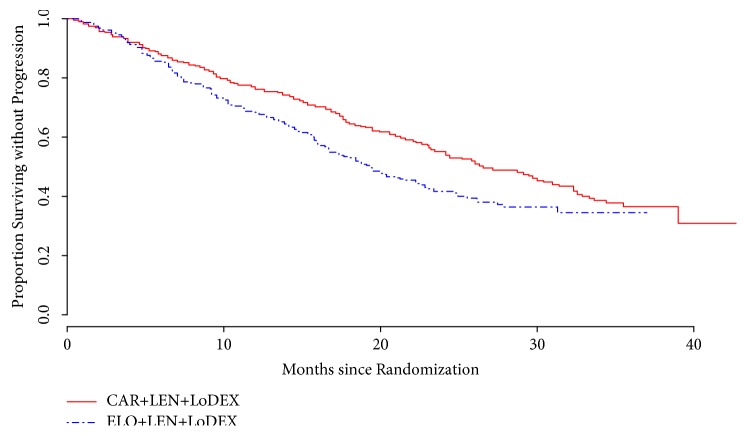
Kaplan-Meier curve of progression-free survival based on reconstructed IPD data. The carfilzomib group and elotuzumab group are compared. CAR: carfilzomib. ELO: elotuzumab; LEN: lenalidomide; LoDEX: low-dose dexamethasone.

As shown in Figure 1, according to the histogram and kernel density of event distribution over time, it seemed that events occurred generally earlier in the elotuzumab group as compared with the carfilzomib group. This was further confirmed by the results of subsequent survival analysis. According to Kaplan-Meier summaries, median PFS was longer in the carfilzomib group (carfilzomib group: median PFS = 26.6 months, 95% CI = [24.2-31.0]; elotuzumab group: median PFS = 19.3 months, and 95% CI = [16.7, 22.8]). According to original study reports, the median PFS was 26.3 and 19.4 months for the carfilzomib group and elotuzumab group, respectively; and the relative errors were only 0.011 and 0.005, respectively. This was consistent with the accuracy evaluation results provided in Guyot and colleagues' work [8] and suggested adequate reliability of the reconstructed IPD data. Further investigation with Cox proportional hazard model revealed significantly better PFS in the carfilzomib group compared with the elotuzumab group (elotuzumab group vs. carfilzomib group: HR = 1.36, 95%CI = [1.11-1.67]). The KM curve of reconstructed IPD data for the two groups is shown in Figure 2. Visually comparing these two curves with those in the original reports, we could not identify any notable difference.

## 4. Discussion

RRMM is the most challenging subtype of MM [4, 5]. Resistance to previous lines of treatment is a big problem [22]. For those with poor response to conventional therapies, participating in a clinical trial involving novel agents or regimens may be the best option to continue fighting against the disease. As a result, RRMM becomes the front line of RCTs which are designed to compare different interventions for MM and produces high quality evidence which can be further used for healthcare stakeholders as important information to be taken into consideration for decision-making. However, RCTs have disadvantages such as the considerable cost in time and resources and the ethical risk that some patients may receive harmful or ineffective treatments during the study. Therefore, any scientific findings providing high quality evidence without much cost should be valued.

In this study, we compared two novel regimens based on published trials, in terms of the PFS which is a very important endpoint in clinical studies. These two triplet regimens both contain lenalidomide and low dose dexamethasone, combined with elotuzumab or carfilzomib. Lenalidomide is one of the second generation IMiDs, which has already been widely used for newly diagnosed MM and RRMM, often in combination with dexamethasone, a glucocorticoid drug with anti-inflammatory and synergistic antimyeloma effects [10, 11, 23]. Elotuzumab is an immune-stimulatory monoclonal antibody which targets signaling lymphocytic activation molecule F7 (SLAMF7) [24]. The expression of SLAMF7 is limited on myeloma and natural killer cells, which ensures minimal effects on healthy tissue [25]. After being evaluated in a phase 2 trial which reported improved PFS [26], the combination of elotuzumab plus lenalidomide and weekly dexamethasone was further evaluated in a phase 3 RCT with lenalidomide and dexamethasone as the control group [19]. The results of this trial indicated that the triplet regimen is better than lenalidomide and dexamethasone alone in terms of PFS. Carfilzomib is a recently developed epoxyketone PI. Carfilzomib exerts significant antimyeloma effect through selectively and irreversibly binding to constitutive proteasome and immunoproteasomes [27]. In a phase 1 and 2 study, carfilzomib, lenalidomide, and low dose dexamethasone combination therapy showed activity in patients with relapsed disease [28, 29]. Recently, a phase 3 RCT comparing lenalidomide and low dose dexamethasone with or without carfilzomibd in relapsed MM patients was completed. The results of this trial also indicated that the triplet regimen is better than lenalidomide and dexamethasone alone in terms of PFS [20].

In order to compare these two arms in terms of PFS, we reconstructed IPD PFS data using established method developed by Guyot et al [8]. According to our results, the algorithm of IPD data reconstruction is reliable with minor error. Survival analysis showed that the carfilzomib group did significantly better than the elotuzumab group in terms of PFS. However, the information in this IPD data set is not abundant, because only three dimensions including the arm, time, and event type (event or censoring) could be reconstructed; as a result, adjustment for covariates could not be performed. Therefore, when interpreting our findings, to which extent this difference in PFS can be attributed to difference in treatment effect between two regimens should be carefully discussed.

We start this discussion by analyzing the baseline difference between these two groups. First of all, the elotuzumab group included patients with refractory or relapsed disease but the cardilzomib group included only relapsed cases. Refractory and relapsed myeloma share many common characteristics in terms of treatment response, and they are usually considered as one individual MM subtype in many clinical trials and guidelines [10, 14, 16–19, 22, 26, 30]. In this sense, this difference may not interfere significantly with treatment effects. As previously mentioned, the proportion of white patients is higher in the carfilzomib group. However, the absolute difference in percentage is only 13%. So far, due to the fact that ethnicity groups other than whites are always underrepresented in trials, no definitive conclusion regarding race and MM prognosis can be drawn [4]. Disease stage has been shown to be correlated with MM prognosis, and advanced disease is generally associated with poor prognosis [31]. According to our results, there are more advanced cases in the carfilzomib group than in the elotuzumab group. Nevertheless, interestingly, the carfilzomib group showed better PFS. From our perspective, the difference in disease stage distribution can be considered as a “plausible confounding” which indirectly demonstrates that the PFS difference should be attributed to effects of other factors, probably the difference of treatment effect between two regimens for the present study. Therefore, according to our results, we think it is very likely that the difference in PFS between these two groups is caused by the difference of treatment effect between the two regimens.

Besides, it should be noted that very interestingly, the two original reports provided similar results that as to PFS, the elotuzumab group or carfilzomib group is better than the common control group which received lenalidomide plus low dose dexamethasone. The HRs of treatment arm versus control arm in these two studies are very close (elotuzumab group vs. control: HR = 0.69, 95% CI = [0.57-0.83]; and carfilzomib group vs. control: HR = 0.70, 95% CI= [0.57-0.85]). From our perspective, this is associated with the different PFS performance of two different control groups; i.e., the control in the carfilzomib trial did better than the control in the elotuzumab trial. And here we came to a very important question, whether the difference in the two control groups was a consequence of difference in baseline characteristics of patients included in these two trials. If the answer is yes, then there might be significant baseline difference between elotuzumab arm and carfilzomib arm, which may interfere with the treatment effect thus make the interpretation of our findings very difficult. We think probably this is not the case. First of all, as previously mentioned, any detected differences in baseline characteristics are either irrelevant or can be considered as plausible confounding. Furthermore, we reviewed the response profile of two control arms and we found the early response endpoints, i.e., the overall response rate of these two control arms, were quite close (66% for the control of the elotuzumab trial and 66.7% for the control of the carfilzomib trial, *P* = 0.81). This indicates that the difference in baseline status between the two study populations did not interact significantly with treatment effect. In other words, this difference probably does not have the power to result in significant difference in efficacy outcomes such as PFS. However, on the other hand, the overall response rate is significantly higher in the carfilzomib arm than in the elotuzumab arm (87% vs. 79%, *P* = 0.003). This difference in response status is consistent with our result in terms of PFS, both indicating that carfilzomib regimen probably has better efficacy than elotuzumab regimen in RRMM patients.

This study has some limitations. Reconstructed IPD data with limited information and minor errors reduced the quality of our results. Unlike analysis with original IPD data, any adjustment for potential confounding factors is impossible. In addition, since neither the mean and standard deviation nor the patient-level data were provided for certain continuous variables including the age, time since diagnosis and number of prior treatment, we could not perform any statistical test to evaluate if there were statistically significant difference between the two groups; besides, evaluation for other baseline characteristics such as cytogenetic risk cannot be performed due to lack of data in the original reports. However, since the primary goal of our study is to investigate the efficacy of two regimens in terms of PFS and any detected differences in baseline characteristics are either irrelevant or can be considered as plausible confounding, the conclusion should be rather reliable.

In conclusion, the CLD regimen may result in better PFS as compared with the ELD regimen in RRMM patients. However, due to limitations of this study, it is recommended to be cautious when applying our findings.

## Figures and Tables

**Table 1:** Regimen used in elotuzumab group and carfilzomib group.

**Arm**	**Regimen**
Elotuzumab group	Elotuzumab: 10 mg/kg on day 1, 8, 15, and 22 for the initial two cycles; 10mg/kg on day 1 and 15 for the following cycles.
	Lenalidomide: 25 mg/kg on days 1-21 for each cycle.
	Dexamethasone: 40 mg orally for the week without elotuzumab; 8 mg intravenously plus 28 mg orally on the day of elotuzumab administration.
	Administered until withdrawal of consent, disease progression, or the occurrence of unacceptable toxic effects.

Carfilzomib group	Carfilzomib: 10 min infusion on days 1, 2, 8, 9, 15, and 16 (starting dose, 20 mg/m2 on day 1 and 2 of cycle 1;target dose, 27 mg/m2 thereafter) for cycles 1-12 and on days 1, 2, 15, and 16 during cycles 13 through 18.
	Lenalidomide: 25 mg/kg on days 1-21 for each cycle.
	Dexamethasone: 40 mg administered on days 1, 8, 15, and 22.
	Administered until withdrawal of consent, disease progression, or the occurrence of unacceptable toxic effects.

**Table 2:** Baseline characteristics of patients.

**Characteristics**	**Elotuzumab group**	** Carfilzomib group**	***P* value**
	**(n=321)**	**(n=396)**	
**Age - yr**			
median	67	64	NC
range	37-88	38-87	

**Male sex - no. (**%**)**	192 (60)	215 (54)	0.16

**ECOG performance status**			
0	159 (50)	165 (42)	0.08
1	138 (43)	191 (48)	
2	24 (8)	40 (10)	

**Race - no.(**%**)**			
White	264 (82)	377 (95)	< 0.0001^*∗*^
Others	57 (18)	19 (5)	

**Time since diagnosis - mo**			
median	41.6	36	NC
range	3.6-208.1	4.8-236.4	

**Disease stage (ISS) no. (**%**)**			
I	141 (44)	64 (16)	< 0.0001^*∗*^
II	102 (32)	99 (25)	
III	66 (21)	185 (47)	
unkown	12 (4)	48 (12)	

**Previous therapy regimens**			
median no.(range)	2 (1-4)	2 (1-3)	NC
regimens no.(%)			
1	151 (47)	184 (47)	0.96
2 or more	170 (53)	211 (53)	

*∗*: statistically significant.

NC: not calculable.

## Data Availability

The data used to support the findings of this study are available from the corresponding author upon request.
